# Time trends in exposure of cattle to bovine spongiform encephalopathy and cohort effect in France and Italy: value of the classical Age-Period-Cohort approach

**DOI:** 10.1186/1746-6148-5-34

**Published:** 2009-09-18

**Authors:** Carole Sala, Giuseppe Ru

**Affiliations:** 1 AFSSA-Lyon, 31 avenue Tony Garnier, 69364 Lyon cedex 7, France; 2 CEA-Instituto Zooprofilatico Spermentale di Piemonte, Ligura e Valle d'Aosta, Via Bologna 148, 10154 Torino, Italy

## Abstract

**Background:**

The Age-Period-Cohort (APC) analysis is routinely used for time trend analysis of cancer incidence or mortality rates, but in veterinary epidemiology, there are still only a few examples of this application. APC models were recently used to model the French epidemic assuming that the time trend for BSE was mainly due to a cohort effect in relation to the control measures that may have modified the BSE exposure of cohorts over time. We used a categorical APC analysis which did not require any functional form for the effect of the variables, and examined second differences to estimate the variation of the BSE trend. We also reanalysed the French epidemic and performed a simultaneous analysis of Italian data using more appropriate birth cohort categories for comparison.

**Results:**

We used data from the exhaustive surveillance carried out in France and Italy between 2001 and 2007, and comparatively described the trend of the epidemic in both countries. At the end, the shape and irregularities of the trends were discussed in light of the main control measures adopted to control the disease. In Italy a decrease in the epidemic became apparent from 1996, following the application of rendering standards for the processing of specific risk material (SRM). For the French epidemic, the pattern of second differences in the birth cohorts confirmed the beginning of the decrease from 1995, just after the implementation of the meat and bone meal (MBM) ban for all ruminants (1994).

**Conclusion:**

The APC analysis proved to be highly suitable for the study of the trend in BSE epidemics and was helpful in understanding the effects of management and control of the disease. Additionally, such an approach may help in the implementation of changes in BSE regulations.

## Background

The first case of Bovine Spongiform Encephalopathy (BSE) was described in 1986 in the United Kingdom, where the disease became noticeable from 1987. In spite of early evidence of the role of meat and bone meal (MBM) in transmission of the disease in the United Kingdom [1,2] and the implementation of the ban on the use of MBM for feeding ruminants, the epidemic extended rapidly to most European countries. In 1996, evidence of BSE's transmission to humans [3,4] led to one of the most important European food crises, underlining the need for Europe-wide regulations to fight the disease. One major challenge was the possibility of extending BSE surveillance in order to estimate the extent of the epidemic in European countries. During 1999, rapid and accurate post-mortem diagnostic tests were developed and introduced for the monitoring of BSE (Amendment of Commission Decision (EC) 98/272). Initial partial testing programmes revealed that BSE prevalence, when based only on the reporting of clinically suspect animals, was largely underestimated. Then in all European countries (European regulation (EC) No 999/2001 and Amendments), from July 2001 on, screening programmes were enforced so that all cattle over 24 months of age (subsequently changed to 30 months), whether slaughtered for human consumption, dead-on-farm or euthanized, had to be tested. The implementation in Europe of this exhaustive surveillance plan meant that the BSE status of countries, an estimate of the extent of the epidemic and its evolution could be updated year by year.

In the last few years, very few BSE cases have been detected in Europe while there has been a downward trend in the disease in all the affected countries [5]. European countries were variously affected by the BSE epidemics. Recent questions have also addressed the comparison of how BSE evolved differently in each country and the role of the successive national and European control measures adopted since the onset of the epidemic.

Various methods have been used to estimate BSE's prevalence and evolution over time [6-10](de Koeijer, Heesterbeek et al. 2004; Supervie and Costagliola 2004; Saegerman, Speybroeck et al. 2006; Prattley, Cannon et al. 2007). Among these methods, the age-period-cohort (APC) models have proved to be well adapted to studying BSE [11,12], as they enable the three main factors influencing BSE prevalence to be taken into account: the age and period at which animals are tested and their birth cohort. Mobilising few hypotheses on disease characteristics and efficiency of control measures, the method should be useful for studying and comparing BSE epidemics trends in different countries.

In our study, we analysed Italian and reanalysed French BSE data in parallel, with a more appropriate data categorisation and method, in order to characterise and compare BSE trends in both countries, as they experienced different types of BSE epidemics. We carried out an APC analysis using two main assumptions: i) the evolution of BSE's prevalence over time was mainly due to the cohort effect, ii) the BSE prevalence of a given cohort was proportional to its exposure to BSE and thus proportional to its BSE risk. We estimated the respective effects of age and birth cohort on the BSE risk, after adjusting for the period when necessary. Using the second differences of the estimated parameters, we have characterised the dynamic of the BSE trends, and examined it in the light of the main control measures adopted to reduce exposure of cattle to the BSE agent in France and Italy.

## Method

### Sources and nature of data

The first French BSE surveillance system, based on reporting of clinically suspect animals in farms and at ante-mortem inspections in abattoirs, was implemented in December 1990. In 1999, the development of rapid tests enabled the implementation of large-scale screening programmes which were first restricted to at-risk animals in 2000 and then extended, from January 2001, to all cattle over 30 months of age entering the food chain. This abattoir screening plan was reinforced with the implementation of an active surveillance plan for fallen stock, so that, from 1 July 2001, in accordance with European regulations (European Regulation (EC) 999/2001) and in addition to the clinical surveillance, all dead cattle over 30 months old were tested in France. The individual data provided by the three surveillance streams were stored in databases maintained by the *Agence Française de Sécurité Sanitaire des Aliments *(AFSSA-Lyon, France) and the *Institut National de la Recherche Agronomique *(INRA Theix, France).

In Italy the active surveillance plan implemented in January 2001 was based on the screening of all animals entering the food chain or dead-on-farm. Subsequently, since the beginning of 2001, all animals over 30 months of age slaughtered and all animals over 24 months of age subject to emergency slaughters or dead-on-farm have been tested. The electronic databases containing the individual data from both surveillance streams were controlled and maintained by the *Centro di Referenza nazionale per le Encefalopatie Animali *(CEA) to which the regional laboratories send analysis results on a monthly basis.

### Data management and assumptions

We used data from the BSE surveillance streams in place since 1 January 2001 and 1 July 2001, dates of the implementation of the exhaustive surveillance in Italy and France respectively. As was previously done for the French BSE analysis [12], data were merged into single database, one for each country, and analysed independently of the surveillance stream.

Animals - especially BSE cases - born or bred outside France and Italy were excluded from the analysis. We considered that such animals may have different feeding histories than those of native cattle, due to specific farming patterns and variations in the implementation of BSE regulations (Table 1). Cases of Atypical BSE were also excluded because of recent knowledge of its epidemiological characteristics compared to those of the classical disease [13-16]. Considering that the threshold age for testing varied during the period of interest from 24 to 30 months, and that no BSE case in cattle younger than 30 months old was detected either in France or in Italy, only animals over 30 months of age were included in the analysis. In the end, 17,247,651 negative-tested animals and 633 classical BSE cases were included in the French database and 4,506,951 negative-tested animals and 131 BSE cases in the Italian database (Table 2).

**Table 1:** Date and content of main control measures enforced in France, Italy and Europe to control BSE.

**Date of implementation**	**European measure**	**Content of the measure**	**Country concerned**
**August 1989**	no	Ban on the importation of MBM from UK	France

**July 1990**	no	Ban on the use of MBM* for bovines	France

**July 1994**	no	Ban on the use of MBM for ruminants	France
			
**August 1994**	Decision 94/381/EC		EU members

**June 1996**	no	Ban on the use of SRM**	France
**April 1997**	Decision 96/449/EC	Standards for batch processing in rendering systems	EU members

**January 1997**	no	Partial SRM ban from BSE-affected countries	Italy
**October 2000**	Decision 2000/418/EC (replacing Decision 97/534/EC that was never enforced)	Ban on the use of SRM	EU members

**January 2001**	Decision 2000/766/EC	Total ban on the use of MBM for farmed animals	EU members

* MBM, Meat and Bone Meal, ** SRM, Specified Risk Material

**Table 2:** Data available and data included in the analysis.

	Data available*		**Data included**		Data categorisation**			Reference groups***		
	**Number of tested animals**	**Number of BSE cases**	**Number of tested animals**	**Number of BSE cases**	**Age** **(year)**	**Birth cohort**	**Period** **(calculated)**	**Cohort**	**Age**	**Period**

**France**	17,306,300	660	17,248,284^(1)^	633^(2)^	2 to 33	1971 to 2005	1999 to 2007	1994/1995	6	2001
**Italy**	4,692,377	141	4,506,951^(3)^	131^(4)^	2 to 12	1988 to 2005	1999 to 2007	1996	5	2001

* data available from the implementation of complete active surveillance in the countries (January 2001 in Italy, July 2001 in France) to 31 December 2007.

** one-year intervals were used to define age groups and birth cohorts. The period was calculated using the relationship: period = age + cohort.

*** the reference groups chosen were the groups with the highest unadjusted prevalence. If necessary, a second model was fitted with the groups with the highest adjusted prevalence as a reference.

^(1) ^58,002 animals with unknown date of birth were removed.

^(2) ^14 atypical cases, three imported cases, nine secondary cases and one case with unknown date of birth were removed.

^(3) ^16,248 animals under two years old, and 19 animals with incorrect age and/or date of birth were removed. 169,156 animals over 12 years old were also removed, because only pooled data for these animals were available (class: "= 13 years").

^(4) ^three atypical cases, three classical cases over 12 years old and four imported cases were removed.

The data were arranged in two-way tables using one-year intervals, with one dimension for the age and the other for the cohort. The period was defined in the diagonal by the linear relation: period = age + cohort and had two-year intervals with a one-year overlap (Additional file 1). As the probability of detecting a BSE case is closely related to the age at which animals are tested [10,17-19], the age in complete years (age at detection for BSE cases and age at screening for negative-tested animals) was systematically included and entered as the first variable in the models. Unlike previous French analyses [12], the birth cohort was defined from 1 January to 31 December, since this method required only the knowledge of the animals' year of birth. Because a significant cohort effect was expected, the cohort was entered as the second variable in the full APC models. Finally, as in previous French analyses, we assumed that the period effect, if any, would mainly be due to variations in the efficiency of surveillance systems and evolution of the diagnostic tests [12]. In both countries there was no indication of relevant changes occurring over time in the respective surveillance systems during the period studied. No strong period effect was thus expected. The period was entered as the last variable in the full APC models.

Lastly, as the total cattle population in both countries was not available, we used the tested cattle population as the denominator to model BSE prevalence at death.

### Measures enforced to control the BSE epidemic in both countries

European and national regulations for both France and Italy were reviewed and listed to describe differences in the time of enforcement for each country. A basic summary of the main measures taken in the two countries is provided in Table 1. The relevant measures were considered to be: (1) the ban on the use of meat and bone meal (MBM) for cattle (*feed ban*) or for all farmed species (*total feed ban*), (2) the measures involving the removal of specified risk material (SRM) from the processing of MBM dedicated to animal feed (*prohibition of SRM use*) and (3) the sterilisation of MBM at 133°C, 3 bar and for 20 minutes (*standards for safe rendering*). Although similar measures were enforced in both countries, France applied them earlier than Italy.

### Analysis

Datasets were analysed with R software [20] using the following logistic model with dummy variables:

(1)

where *a*_*i*_, *c*_*j *_and *p*_*k*_, (*k *= *i*+*j*-*I*) were the dummy variables for age, cohort and period, *α*_*i*_, *β*_*j *_and *γ*_*k *_were the fixed effects for i^th ^age group, j^th ^cohort and k^th ^period respectively, *P*_*ijk *_the expected prevalence of BSE in the group of age i, cohort j and period k, *ε*_*ijk *_the stochastic error and *P*_0 _the log of the odds of the reference group. Reference groups were defined as the categories with the highest unadjusted prevalence so that *α*_6 _= *β*_1995 _= *γ*_2001 _= 0 for the French data and *α*_5 _= *β*_1996 _= *γ*_2001 _= 0 for the Italian data (Table 2). For the French data, a second model was fitted with the cohort with the highest adjusted prevalence as a reference to facilitate interpretation of the results (*α*_6 _= *β*_1994 _= *γ*_2001 _= 0).

To perform the APC analyses of each dataset, we used, as previously [12,21], the stepwise procedure recommended by Clayton and Schiffler [22,23] (Table 3) and fitted the full APC models by equalising the effect of two successive periods so that *γ*_2001 _= *γ*_2002_.

**Table 3:** List of the APC models fitted and selection of the best models. Quality of fit and contribution of each new variable added to the model are presented.

		**Adjustment of the models**			**Estimate of the effect of the covariates**				
**No.**	**Model**	**Residual deviance**	**df**	p-value*	**Comparison with model**	**Difference of deviance**	**Difference of df**	p-value**	**Tested effect**

	**France**								
**0**	Null	1958.7	232	0.000					
**1**	Age	991.8	201	0.000	**0**	966.9	31	0.000	Age
**2**	Age-Drift	344.3	200	0.000	**1**	647.5	1	0.000	Drift^(1)^
**3a**	Age-Cohort	68.0	167	1	**2**	276.3	33	0.000	Non-linear cohort effect
**3b**	Age-Period	336.4	194	0.000	**2**	7.9	6	0.246	Non-linear period effect
**4**	Age-Cohort-Period^(2)^	55.5	161	1	**3a**	12.5	6	0.051	Period effect (non-linear + linear)
	**Italy**								
**0**	Null	347.4	91	0.000					
**1**	Age	224.9	81	0.000	**0**	122.5	10	0.000	Age
**2**	Age-Drift	101.4	80	0.053	**1**	123.6	1	0.000	Drift^(1)^
**3a**	Age-Cohort	49.6	64	0.907	**2**	51.8	16	0.000	Non-linear cohort effect
**3b**	Age-Period	90.3	73	0.082	**2**	11.1	7	0.134	Non-linear period effect
**4**	Age-Cohort-Period^(2)^	32.5	57	0.996	**3a**	17.1	7	0.017	Period effect (non-linear + linear)

* goodness of fit test, ** log-likelihood ratio test (α = 5%), for p > 0.05 the effect of the variable is non-significant

df, degree of freedom

^(1) ^linear effect of the period and cohort combined

^(2) ^period is the last covariate entered in the model

The significance of the effect of each new variable added to the model was assessed by the log-likelihood ratio test (Table 3). The goodness of fit was evaluated by an examination of the residual deviance of the models, which approximately follows a Chi-square distribution [24].

In addition, we calculated the second differences on a log-scale:

(2)

(3)

to estimate the local curvature at specific points for the age and cohort. For the period, because of its overlapping intervals, we used the average of adjacent second differences:

(4)

**Figure 1:**
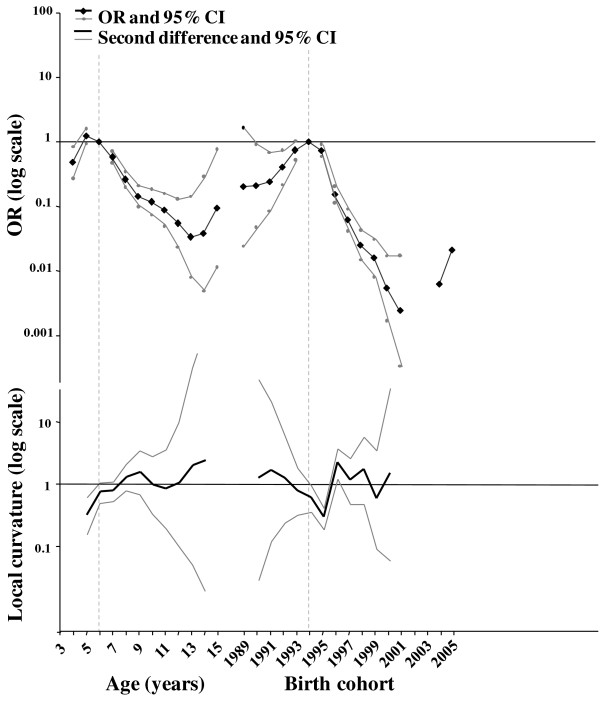
**Results of the AC modelling of the French data and second differences**. The effects are expressed in OR with 95% CIs, plotted on a log scale (null values and very large CIs are not represented) and correspond to the BSE risk of a specific age group and birth cohort relative to the reference age group and the reference birth cohort (respectively five years and 1994 and indicated using dotted light grey vertical lines on the graph). To facilitate interpretation, the second differences on the logarithmic scale were plotted as corresponding contrasts (local curvatures).

**Figure 2:**
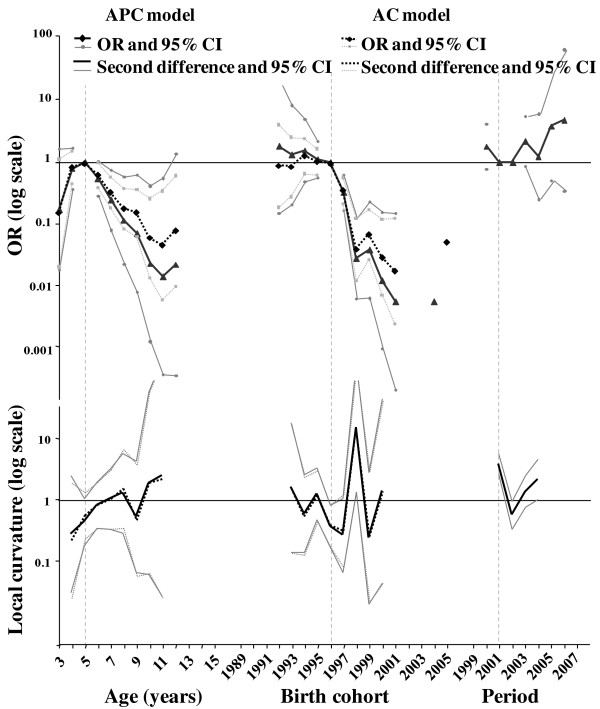
**Results of the APC and AC modelling of the Italian data and second differences**. The effects are expressed in OR with 95% CIs, plotted on a log scale (null values and very large CIs are not represented) and correspond to the BSE risk of a specific age group and birth cohort detected at a specific period relative to the age, birth cohort and period reference groups (respectively five years, 1996 and 2001 and indicated using dotted light grey vertical lines on the graph). To facilitate interpretation, the second differences on the logarithmic scale were plotted as corresponding contrasts (local curvatures).

To facilitate interpretation, the second differences were plotted on a logarithmic scale as the corresponding contrasts (local curvature in Figures 1 and 2) between adjacent categories: a contrast of one indicates a constant trend, an estimate of more than one indicates acceleration and finally a contrast estimate of less than one is associated with an attenuation of the trend in prevalence [23].

## Results

Effects of variables were expressed as the Odd Ratio (OR) with 95% confidence intervals (CIs). In the case of BSE, the OR could be considered as a good approximation of the relative risk (RR) of BSE (subsequently referred to in the text as the BSE risk) i.e., the risk for an animal of age *a*, birth cohort *c *and tested at period *p*, of being a BSE case compared to the same risk for an animal in the reference groups, in which OR = 1.

### French data

The results are presented in Table 3 and Figure 1. According to the goodness of fit and the log-likelihood ratio tests, the best model was the age-cohort (AC) model, with a highly satisfactory fit (p = 1). Unlike previous French analyses [12], adding the period effect did not improve the model further (p = 0.052).

However, results for age and cohort effects did not differ from those previously obtained. Five- and six-year-old animals had the same highest risk of BSE, while animals older than seven years and younger than 16 years presented a significantly lower risk as well as four-year-old animals. Concerning the BSE risk for the birth cohort, animals born in 1993 and 1994 showed the highest risk while animals born in 1990, 1991 and 1992 had a lower, but increasing, BSE risk. There was a clear decline in risk for animals born in and after 1995. It was not possible to draw any conclusions for animals born before January 1990 and after January 2002, either because CIs included 1 or no estimates were available.

The examination of second differences for the cohort effect showed significant irregularities in the BSE risk trend around the 1994, 1995 (deceleration) and 1996 (acceleration in trend) birth cohorts.

### Italian data

Although the two BSE epidemics showed a similar decline over time, the results of the APC analysis for the Italian data (Table 3 and Figure 2) differed from those of the French data. The best model was the full APC model with a very good fit (p = 0.996) and statistically significant period effect (p = 0.017). However, the AC model also had a very satisfactory goodness of fit (p = 0.907) and showed similar results for age and cohort effects (Figure 2).

Despite the fact that the period effect was significant, the BSE risk (in terms of OR) did not differ from one observation period to another, since all CIs included 1. However, the second differences showed two irregularities around the periods 2001 (acceleration) and 2002 (deceleration of the trend) in which the effect of the period deviated from the overall flat linear trend.

Considering the age effect, although the BSE risk did not differ significantly for the three- to six-year-old animals, an increasing risk from three to five years is suggested, whereas animals from seven- to 11-year-old included, presented a significantly lower risk. For the cohort effect, animals born from January 1992 to December 1995 had the same higher risk of BSE compared to that of animals born in 1996 (no clear peak as in France was evident). An initial apparent decreasing trend was evidenced for animals born after 1995. Afterwards the risk decreased steadily for animals born from January 1996 to December 2001 with the exception of animals born in 1999. In addition, the examination of second differences showed irregularities around the 1996 (deceleration) and 1998 (acceleration in trend) birth cohorts.

## Discussion

Using APC modelling to study the time trend of BSE epidemics, we assumed that the trend of the disease was mainly related to the cohort effect itself, due to the control measures adopted to reduce the BSE exposure of cattle. We expected that the irregularities of the time trend for the cohort effect, corresponding to the curvatures, could be in connection with the main national and European measures implemented in France and Italy to control the disease.

### Method and assumptions

We used a classical APC categorical analysis with one-year data categorisation. Such categorisation of the data (calculation of the period variable and time span) was based on the same assumptions as those discussed in previous studies [12,25-27].

The use of a categorical analysis for continuous variables was strongly criticised, as it was noted that such a method was not appropriate [26,28-31]. In previous studies, we discussed the respective advantages and disadvantages of categorical and continuous APC analyses [12]. Instead of applying the function to continuously model the effect of age, cohort and period effect, we preferred a categorical analysis to compare French and Italian BSE epidemics. Indeed, the introduction of the categorical analysis was easier, as it did not require the choice of functional form to model the effect of the variables, nor the selection of knots, needed by spline regression. We estimated that the categorical approach, even if it suffered from a lack of precision and/or performance in estimating the effects of the variables - especially for extreme categories - would be sufficient to compare global BSE trends. In addition, and where we previously used knots when spline modelling, we used second differences to estimate trend changes in the effects of the variables. We assumed that the examination, for the cohort effect, of global trends and second differences combined, may help in estimating when the control measures became effective and their impact on the disease trend. Additionally, second differences indicating local changes around a specific time did not depend on the constraint used to identify the model [23].

The identification of the full APC model was based on the same assumption as those used in previous French analyses. Firstly, a significant cohort effect was expected. We assumed that i) feeding was the major, if not only, route of BSE infection for cattle [1,32,33] and ii) the successive control measures adopted against BSE played the main role in reducing the infectivity of feedstuff over time. Conversely, we expected no or very little period effect as no known change occurred in the surveillance system in either country during the time period of interest. Thus, to identify the full APC model, we used the minimal additional constraint [34] and equalised the effect of two successive periods. We assumed that constraining the parameters of the period would have had a minimal impact on the estimation of the age and cohort effects. Results obtained confirmed the accuracy of our hypothesis regarding primary age and cohort effects.

### Results and characterisation of the effect of control measures

The non-linear period effect identified in the Italian analysis could be an artefact due to the design of the study. The significant acceleration of the trend around the 2001 period (second differences over 1) could be related to the particular categorisation of the data which artificially created 1999-2000 periods and discrepancy between the prevalence of the 1999 and 2001 periods. A similar unexpected period effect had already been identified in previous French APC analyses [12,21]. The lack of a period effect in the current French analysis, where data categorisation differed from that of previous studies, favoured the artefactual nature of the period effect. However, although we could not completely exclude a minor real period effect in connection with a change in sensitivity of diagnostic tests over time [35,36], such a period effect did not impact on the estimation of the age and cohort effects.

Thus, reanalysing and analysing French and Italian datasets confirmed that the peak of the BSE risk involved animals aged five to six years at testing. This result was consistent with previous estimates of a very young age at infection (under one year of age) and an incubation period of around five years [10,18,37].

In line with our hypotheses and previous studies, the models evidenced a strong cohort effect in both countries [37,38]. The results of the French analysis were in line with those previously obtained with the same model or different approaches [7,12,37,39]. The first significant increasing BSE risk trend was evidenced for the 1990-1993 birth cohorts. As discussed in the spline analysis of French data, this increase followed an initial peak of infection and was probably linked to cross-contamination and recycling of infectious material, demonstrating the inefficiency of the initial control measures adopted in 1989 and 1990 in France. Then, the "second" peak of exposure - and the only one we evidenced in our analysis - was reached for animals born in 1993 and 1994. Considering the one-year delay in implementation of the control measure, the extension of the MBM ban to all ruminants (1994) coincided with the start of the decline of the epidemic (1995). The deceleration in trend around the 1995 birth cohort could be connected with the inversion of the trend at the peak, while the acceleration in the declining trend in 1996 might be related to the enforcement of the ban on the use of SRM and (after few months) of the new standards for rendering systems. These results were in accordance with results obtained with the spline method, in which selected models indicated curve changes in 1995 and 1996.

With regard to the results of the Italian analysis, the pattern of the BSE risk for birth cohorts appeared to be similar to that of the French data but with a one-year time delay, which is similar to the delay that occurred in the application of the control measures. Unlike the French results, no significant BSE risk peak was evidenced. Because of the few cases available for analysis, the declining pattern in the trend between 1992 and 1995 showed very large confidence limits for the annual estimates. The feed ban implemented in 1994 did not result in a steady decline as suggested by the deceleration in the trend in 1996, and this is consistent with previous studies that suggested the potential role of cross contamination in feed mills [38]. Only the combined effect of the 1997 partial SRM ban and the improvement in rendering standards helped accelerate the declining trend. The temporary peak in the 1999 cohort may also suggest that the partial SRM ban was only partially effective.

To date, the number of BSE cases diagnosed in the most recent cohorts is very small and testing of those cohorts is still incomplete: therefore, our analysis did not enable us to assess the effectiveness of the most recent European control measure, i.e., the total MBM feed ban enforced since January 2001. However, the few cases recently detected have mostly been in animals born in the 1990s and so far, only two BSE cases in cattle born after 1 January 2001 have been detected in France and Italy (one French animal born 1 January and one Italian animal born in January 2001[40]).

The classical categorical approach combined with the examination of second differences gave similar results to those obtained with the use of regression splines in modelling the functional form of the covariates. Easier to implement than the continuous method, categorical analysis has enabled a comparison of the time trends experienced by different populations. Currently our approach is being extended to a wider range of countries affected by the BSE epidemic [41].

## Conclusion

As shown, the APC approach is, in general, particularly appealing when studying time trends in health problems affecting populations that may have very long latencies and in which the age of individuals, the cohort and/or the year of diagnosis are important factors to take into account. Therefore, the APC analysis was highly suitable for the study of the time trend of a disease such as BSE in cases where adequate hypotheses make it possible to disentangle the effects of the three main factors (i.e. age at diagnosis, birth cohort and period of animal testing) affecting the prevalence of BSE.

## Authors' contributions

CS collected the French data, performed the data analyses and drafted the manuscript. GR collected the Italian data, supervised the data analyses and participated in the drafting of the manuscript. Both authors read and approved the final manuscript.

## Appendix

The APC models were firstly developed in demographic sciences where they were practical tools for interpreting mortality data. They proved to be well-adapted to all topics in which the age of individuals at the time of the event, their birth cohort and the observation period were influencing factors [27,42]. Then, ever since Frost [43] introduced APC models to epidemiology, they have played a crucial role in epidemiological studies, where they are still routinely used for time trend analysis of incidence or mortality rates [44-50]. In this matter, the aim of the APC analysis is to estimate the respective effects of age, period (calendar time) and cohort (birth time) on the variable of interest, either the specific mortality rate, or the incidence or prevalence of a disease. In the APC models, age is considered as being the most important influencing factor since it plays a major role in the occurrence of most diseases. While age is associated with inherent biological processes, the observation period is associated with external factors that have an equal and simultaneous effect on all the cohorts and ages at a specific period of time. In these models, the third variable, the birth cohort, is traditionally made up of a set of individuals sharing common experience so that the cohort effect is presumed to reflect all the events that affect all the individuals of a cohort equally, independently of their age and observation period [51,52].

Classically, before using any formal APC analysis, incidence data - i.e. number of cases and person-years as the denominator - are arranged in a two-way contingency table, better known as the Lexis diagram, with one dimension for the age and the other for the period, the cohort being defined by the diagonal of the diagram [27,42]. It is recommended to group data using the same interval width (e.g. one-, five- or 10-years) for the two main dimensions while the third dimension is calculated from the two others using the linear algebraic relationship: cohort = period - age. This way of identifying the third variable leads to an overlap of its intervals which are double those of the other variables (Additional file 1).

**Figure 3:**
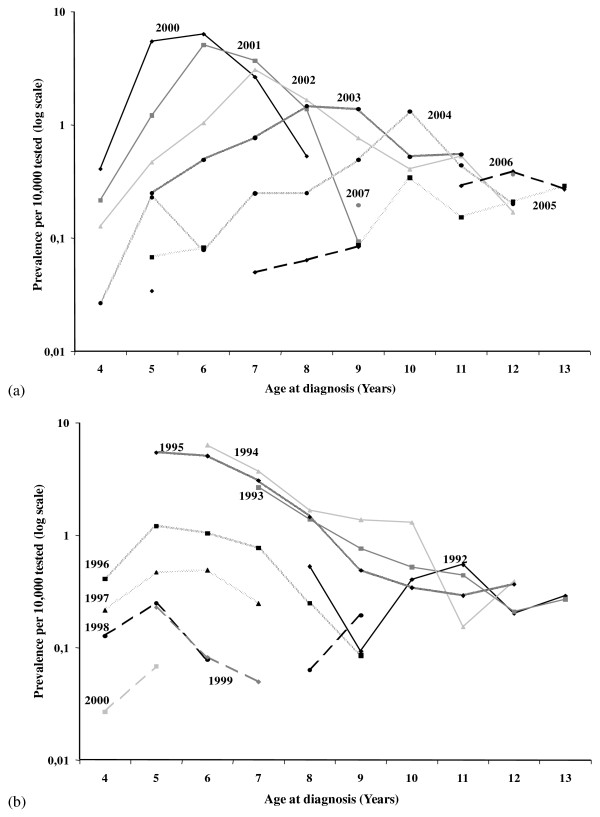
**The classical plots for observation data of the Lexis diagram in the case of BSE in France**. BSE prevalence per 10,000 tested animals in the 2001-2007 period (calculated period): (a) age-specific prevalence per period of diagnosis; (b) age-specific prevalence by birth cohort.

Originally, before the development of the analytical approach, the APC method was essentially based on a graphical analysis of the plots of the observed rates of the Lexis diagram to describe the effect of age, period and cohort. Despite the fact that the most common approach is currently based on statistical methods, it is still recommended to plot such graphs before carrying out any analysis (Figure 3) [25,38,53]. This ensures that the categorisation of the data is adequate for obtaining stable rates across age groups, periods and cohorts and that the hypothesis of the additive effect of the model's variables is correct [54]. The most common approach to analysing the data, even if it is not the best one for continuous variables [29], is to fit categorical log-linear Poisson regression models with one parameter per level of age, cohort and period. The following model is also used, assuming the effect of additive variables on the log-scale:

(1a)

where λ_*ijk *_is the disease rate of the group of age *i*, period *j *and cohort *k*, λ_0 _an intercept, *a*_*i*_, *p*_*j *_and *c*_*k *_(where *k *= *j*-*i*+*I*) the dummy variables for the age, period and cohort, α_*i *_*β*_*j *_and γ_*k *_the fixed effects for the i^th ^age group, j^th ^period and k^th ^cohort and ε_*ijk *_the stochastic error. However, such a model is not identifiable since an over-parametrisation of the model occurs when the three variables are simultaneously entered in it, due to the exact linear dependency between the age, period and cohort. This non-identifiability problem of the full APC model is an old, well-known problem and, despite many suggestions, the question has still not been satisfactorily solved. The usual constraint, consisting in taking one level of age, period and cohort as a reference, i.e. equalising one parameter of each variable to 0, is not sufficient for identifying the model. The model is not capable of differentiating the respective effects of the age, period and cohort. Considering that the effect of each variable could be separated into linear (trend or drift) and non-linear parts (curvature), the linear components of the effects of the covariates cannot be estimated altogether [34,54,55].

An attractive solution could be to use two-factor models, i.e. AC or AP models, in which the estimates are possible. However, it was pointed out that these models require strong assumptions with regard to the non-effect of the removed variable and therefore, this solution may be not appropriate in many situations. Using interactions in two-factor models was also proposed, as the period and cohort could be considered as the interaction between age and cohort and age and period respectively. However it was noted that such interactions were very specific forms of interactions whose effects were not all accessible and complicated to interpret [51,54].

Nevertheless, it has been demonstrated that a minimal additional constraint, equalising to 0 the effect of two levels of one of the covariates,

(2a)

is sufficient for identifying the model [34,56]. However, such a constraint is often arbitrary and each different additional constraint, used on the same dataset, may provide completely different estimates of the effects of the variables, despite the same quality of fit, so that it is difficult to know which model is the most appropriate [34,54,55,57]. Using multiple constraints was then proposed, i.e. equalising more than two levels of one or more variables. Under these conditions, if the different sets of constraints used are still providing different estimates of the parameters, different degrees of fit are also provided, so that it is possible to estimate which model is the most appropriate one [34]. Such a process tends to be a stepwise procedure, successively removing one or two variables from the model by equalising all its parameters to 0.

Later, another stepwise procedure was proposed by Clayton and Schiffler [23]. Considering that the effect of each variable included both linear and non-linear components, they based their approach on the concept of an overall "drift" - or global trend - due to the undistinguished linear effects of the period and cohort. Assuming the primacy of the effect of age on disease, they advised successively fitting age-model (A), age-(period or cohort) drift model (B), age-period (C1) and age-cohort (C2) models and, if any of them fit the data correctly, the full APC model (D). With such a process, comparing the fit of model B (in which the effect of the period and cohort are only linear), with models C1 or C2, allows an estimation of the significance of the non-linear effects of the period and cohort respectively. The comparison between models C1 or C2 with model D tests for the cohort effect (linear and non-linear) adjusted for the period and vice versa.

Finally, other strategies were developed around the part of the model that could be estimated without bias, i.e., which would not depend on the additional constraint used to fit the model [23,53,55,58,59]. Separating the effect of each variable into linear and curvature parts, it was demonstrated that the curvatures, which could be considered as a deviation from the linearity of the effect, and the global slope (net-drift), i.e. the sum of the slopes of two variables, were constant whatever the minimal additional constraint used. However, it was pointed out that the global slope does not enable the discrimination of the respective linear effects of each variable and that the curvatures are not easy to interpret. Nevertheless, Clayton and Schiffler suggested using local curvature which could be expressed as contrasts between relative risk (RR) of adjacent levels of a same variable so that the curvature in *age i *was:

(3a)

These identifiable contrasts determine the curvatures of secular trends, i.e. they represent a measure of the acceleration of the trend around age *i*. On the log scale, these contrasts corresponded to second differences:

(4a)

A zero value means that the log-risk of the age *i *is locally a straight line (i.e. absence of change in the local trend). Positive or negative values indicate respectively convex or concave relationships [23], i.e. respectively a sharp deceleration or a sudden acceleration in the trend associated with the age *i.*.

In summary, many suggestions were made on how to handle the non-identifiability problem of the full APC model, while no ideal solution was found. Moreover, despite the fact that the choice of the additional constraint has a major impact on the observed pattern for the estimates of the age, period and cohort effects, this method is a very common way of identifying the role of the three variables in the trend, in particular when we have *a priori *knowledge of the specific effect of one of the three variables. Often, the combining of the stepwise procedure proposed by Clayton and Schiffler and the minimal additive constraint chosen on biological or epidemiological knowledge, is an acceptable solution for performing an APC analysis in major epidemiological studies.

## Supplementary Material

Additional file 1 **Overlapping intervals of the calculated period**. Animals aged five years (in complete years) and from the 1996 cohort (born from 1 January 1996 to 31 December 1997) were tested from 1 January 2001 to 31 December 2002 and are thus included in the 2001 period (1996 + 5 = 2001); animals aged five years (in complete years) and from the 1997 cohort (born from 1 January 1997 to 31 December 1998) were tested from 1 January 2002 to 31 December 2003 and are included in the 2002 period (1997 + 5 = 2002) and so on. Click here for file
