# Supplementary material for: Time trends in exposure of cattle to bovine spongiform encephalopathy and cohort effect in France and Italy: value of the classical Age-Period-Cohort approach
Source: BMC Vet Res. 2009 Sep 18;5:34. doi: 10.1186/1746-6148-5-34 (PMC2758858; doi:10.1186/1746-6148-5-34)
Supplement: Additional file 1 — Overlapping intervals of the calculated period. Animals aged five years (in complete years) and from the 1996 cohort (born from 1 January 1996 to 31 December 1997) were tested from 1 January 2001 to 31 December 2002 and are thus included in the 2001 period (1996 + 5 = 2001); animals aged five years (in complete years) and from the 1997 cohort (born from 1 January 1997 to 31 December 1998) were tested from 1 January 2002 to 31 December 2003 and are included in the 2002 period (1997 + 5 = 2002) and so on. [file 1746-6148-5-34-S1.DOC]

| **COHORT** | | **AGE** | **PERIOD** | | | | | | | | | |
| --- | --- | --- | --- | --- | --- | --- | --- | --- | --- | --- | --- | --- |
|  | |  | **2001** | | **2002** | | **2003** | | **2004** | | **2005** | |
| Jan | Dec |  | Jan | Dec | Jan | Dec | Jan | Dec | Jan | Dec | Jan | Dec |
| **1996** | | **5 years; [60-72[ months** |  | **2001** | |  |  | | | | | |
|  | |  |  | | | | | | | | | |
| **1997** | | **5 years; [60-72[months** |  | | **2002** | | | |  | | | |
|  | |  |  | | | | | | | | | |
| **1998** | | **5 years; [60-72[ months** |  | | | | **2003** | | | |  | |
|  | |  |  | | | | | | | | | |
| **1999** | | **5 years; [60-72[ months** |  | | | | | | **2004** | | | |
